# Dynamical proxies of North Atlantic predictability and extremes

**DOI:** 10.1038/srep41278

**Published:** 2017-01-25

**Authors:** Davide Faranda, Gabriele Messori, Pascal Yiou

**Affiliations:** 1 Laboratoire des Sciences du Climat et de l’Environnement, LSCE/IPSL, CEA-CNRS-UVSQ, Université Paris-Saclay, F-91191 Gif-sur-Yvette, France; 2 Department of Meteorology, Stockholm University and Bolin Centre for Climate Research Science, Stockholm, Sweden

## Abstract

Atmospheric flows are characterized by chaotic dynamics and recurring large-scale patterns. These two characteristics point to the existence of an atmospheric attractor defined by Lorenz as: “the collection of all states that the system can assume or approach again and again, as opposed to those that it will ultimately avoid”. The average dimension *D* of the attractor corresponds to the number of degrees of freedom sufficient to describe the atmospheric circulation. However, obtaining reliable estimates of *D* has proved challenging. Moreover, *D* does not provide information on transient atmospheric motions, such as those leading to weather extremes. Using recent developments in dynamical systems theory, we show that such motions can be classified through instantaneous rather than average properties of the attractor. The instantaneous properties are uniquely determined by instantaneous dimension and stability. Their extreme values correspond to specific atmospheric patterns, and match extreme weather occurrences. We further show the existence of a significant correlation between the time series of instantaneous stability and dimension and the mean spread of sea-level pressure fields in an operational ensemble weather forecast at lead times of over two weeks. Instantaneous properties of the attractor therefore provide an efficient way of evaluating and informing operational weather forecasts.

Dynamical systems analyses have led to the crucial notion that atmospheric motions are chaotic and settle on an attractor^1^. Estimates of the average dimensions *D* of atmospheric attractors were produced from times series of various meteorological variables^2^, because this quantity roughly indicates the numbers of degrees of freedom sufficient to describe the flow in its average, time-stationary configuration. However, many weather phenomena of great societal and economic relevance such as extratropical storms, heatwaves and cold-spells are linked to transient metastable states of the atmosphere, whose dynamical properties depend on the instantaneous rather than average properties of the attractor^3^. Such local properties are uniquely determined by two quantities: the local dimension and stability of the state being considered^4^.

The concept of instantaneous dimension is intuitive: for a state *ζ* of the attractor (an atmospheric configuration), the instantaneous dimension *d*(*ζ*) measures the density of neighbouring points (similar configurations). This implies that *d* can be related to both the entropy and the predictability of nearby trajectories^5^. The stability of the state *ζ* is measured by *θ*(*ζ*) defined as the inverse of the average persistence time of trajectories around *ζ*. If *ζ* is a fixed point of the dynamics, *θ*(*ζ*) = 0. For a trajectory that leaves the neighbourhood of *ζ* immediately, *θ* = 1. In general, the more persistent the configuration *ζ*, the longer the previous and subsequent states of the system will resemble *ζ*.

These instantaneous properties have not been previously computed for atmospheric flows for two main reasons: (i) until recently the length of high-frequency, geographically extensive meteorological time series has been insufficient to allow reliable estimates, (ii) the methodologies used to compute the average dimension of the attractor were not suited to the purpose. In this paper we compute the distribution in phase space of the instantaneous dimensions 0 < *d* < ∞ and the inverse of the persistence 0 < *θ* < 1 for daily sea-level pressure (SLP) fields in the North Atlantic. We apply a novel methodology based on the universal behavior of the Poincaré recurrences in chaotic systems and exploit its link with extreme value theory to estimate *d* and *θ*^4^. The salient features of the methodology are that (i) it removes major uncertainties associated with past estimates^2^,^6^ (see Methods), and (ii) it yields the full probability distribution of the instantaneous dimension of the attractor. This approach is validated on the Lorenz idealized system^7^ and a high-dimensional random system (see Methods and Extended Data Fig. A.1).

We chose SLP as a representative field for the large-scale atmospheric circulation over the North Atlantic and Europe (see Methods). To verify the robustness of our results we analyse two distinct daily SLP timeseries using NCEP/NCAR^8^ and ERA-Interim data^9^. The average dimensions *D* obtained by averaging *d* over all *ζ*, including tests performed on coarse-grained NCEP/NCAR data, are shown in Fig. 1-a. If we interpret the resolution, which is of order 10^4^ grid-points for ERA-Interim and of order 10^3^ grid-points for NCEP/NCAR, as an upper bound for *D*, our results point to the existence of a low dimensional attractor. The values of *D* are comparable across all resolutions, except when the coarse graining degrades the resolution to the point where large-scale SLP low and high centers become unrecognizable (resolution 20°) and the phase space itself is shrinking. We find that the distribution of *d*(*ζ*) for the 2.5° NCEP/NCAR reanalysis ranges from as low as 3 to as high as 20 (Fig. 1-b). The average value *D* = 13 is only representative of a limited number of daily pressure fields.

Our estimate of *D* is not the dimension of the global climate attractor: the dynamics followed by our attractor is representative only of large-scale pressure structures (cyclones or anticyclones) but is practically insensitive to smaller scale phenomena. The large spread in the distribution of instantaneous dimensions (3 < *d*(*ζ*) < 20) explains why deterministic low-dimensional models are unable to reproduce the transients between metastable states of the atmospheric circulation, such as the transitions between zonal and blocked phases of the mid-latitude flow^1^,^3^.

Figure 1-c displays the time series of *d*(*ζ*). A seasonal cycle is identifiable both from the whole time series and from the inset which shows the last three years of instantaneous dimension estimates. The troughs occur in summer and the peaks in winter, when the temporal variability of the instantaneous dimension is also high. Similar considerations hold for *θ* (Extended Data Fig. A.2) and for the ERA-Interim datasets (Extended Data Fig. A.3). The linear correlation coefficient between the ERA-Interim and NCEP series of *d* is 0.47, while it is 0.61 for *θ*.

We now use *d*(*ζ*) and *θ*(*ζ*) as probes to investigate the large-scale dynamics and associated weather extremes of the North Atlantic. Here we focus on the highest resolution NCEP/NCAR data (see Extended Data Figs A.4 and A.5 for the ERA-Interim results). The scatter plot of *d* versus *θ* (Fig. 2) shows that these variables are correlated: when the dimension is high, the trajectory is an unstable region of the phase space. It is then unlikely that the trajectory sticks for long time around a certain point. Conversely, the trajectory is confined to a smaller space for low dimensions, with an increasing probability of being there at the next iteration. The analysis of idealised systems, such as the one proposed by Lorenz^7^, suggests that extremes in *d* and *θ* correspond to extremes in phase space (see Extended Data Fig. A.1 and Methods).

We begin by isolating all the days *t* such that the instantaneous dimension *d* and persistence *θ* are beyond the 0.02 and 0.98 quantiles of the respective distributions. The results are insensitive to the exact choice of quantile. The North Atlantic SLP experiences a strong seasonal cycle with significant changes in the principal atmospheric patterns and modes of variability between the winter and summer seasons (e.g. ref. 10). If extremes in the instantaneous properties have a direct correspondence to the large-scale circulation, a similarly pronounced seasonal cycle in their occurrence might be expected. Indeed, the dimensional extremes occur almost exclusively during the extended boreal winter period, as do the maxima of *θ* (Fig. 3). The *θ* minima, on the opposite, occur from early summer into winter. Since these are the most persistent patterns, the summertime episodes might be linked to the more stable dynamics of the atmosphere during this season.

The SLP anomaly patterns corresponding to the extremes in the instantaneous dimension *d*(*ζ*) and persistence *θ*(*ζ*) are shown in the four side panels of Fig. 2. As expected, they correspond to very large SLP anomalies. More surprisingly, all four composites resemble well-known large-scale weather regimes^11^,^12^,^13^. The persistence of the patterns ranges between three days and just over one day (0.3 < *θ* < 0.8). This should not be compared directly to the persistence of the traditional weather regimes, as the requirement that the flow does not leave the neighbourhood of the state *ζ* is a more restrictive condition than that imposed by clustering algorithms.

The minima in daily dimension correspond to positive North Atlantic Oscillation (NAO) conditions, while the minima in *θ*(*ζ*) match negative NAO conditions. A positive NAO favours the occurrence of cyclones across the North Atlantic^14^ and destructive surface winds over continental Europe^15^. Indeed, we find a match between low instaneous dimensions and historical storms: the names and dates of storms corresponding to minima of *d*(*ζ*) are reported in Fig. 4 (see also Methods). Both NAO phases also have a strong impact on the downstream temperature extremes over Europe^16^. However, we note that there are differences between the impacts of the traditional weather regimes and the patterns we identify here (see Extended Data Figs A.6, A.7 and Methods). Both the largest and the smallest NAO values mostly display below-average *d*(*ζ*), thus affording good predictability in accordance with recent analyses of ensemble prediction systems^17^. The maxima of *θ*(*ζ*) and *d*(*ζ*) are both associated with a blocked zonal flow and resemble the Atlantic Ridge and the Blocking regimes, respectively. These are linked to European temperature extremes, although our patterns again display some novel connections (see Extendend Data Figs A.6, A.7 and Methods). Blocked zonal flows are notoriously difficult to forecast^17^. We interpret this as reflecting the transition from persistent low instantaneous dimensions to less persistent, higher dimensions as the atmosphere shifts from a zonal NAO-type flow to a blocked configuration.

We argue that these results can be used in an operational forecast system. If our dynamical indicators are indeed linked to predictability, given a certain state they should correlate with the skill of the model in predicting the future states of the field. Here we partially explore this possibility – because we do not compute the full attractor of the climate model – by correlating the values of *d* and *θ* for the period 2000–2015 with the output of the 2^nd^ Generation NOAA Global Ensemble Reforecast data set^18^. This is consistent with the current operational NCEP Global Ensemble Forecast System, and consists of 11 ensemble members. The ensemble spread at different forecast hours is defined as: 〈*σ*_*SLP*_〉, where angular brackets indicate spatial averages over the region considered in the analysis. The correlation coefficient *R* is significant at all forecast lead times at the 0.05 significance level, reaching *R*(*θ*, 〈*σ*_*SLP*_〉) = 0.42 and *R*(*d*, 〈*σ*_*SLP*_〉) = 0.2. This correlation is partly due to the presence of similar seasonal cycles for the three variables, but remains significant even when the seasonality is removed (see Extended Data Figs A.8 and A.9). Figure 5-a,b shows the bivariate histograms of (*d*, 〈*σ*_*SLP*_〉) and (*θ*, 〈*σ*_*SLP*_〉) for forecasts initialized on all days in the data set and a lead time of 384 hours, just beyond the two weeks predictability limit conjectured by Lorenz^7^ and widely analysed by Kalnay^19^. There is a strong linear relationship between *θ* and 〈*σ*_*SLP*_〉, although the histogram suggests that the distribution is not uniform and peaks at either at low (〈*σ*_*SLP*_〉 ≃ 400 Pa) or high (〈*σ*_*SLP*_〉 ≃ 800 Pa) values. This latter feature might be dependent on the reforecast product. The analysis further suggests that *θ* is a better proxy of ensemble spread 〈*σ*_*SLP*_〉 than *d*. The ensemble spread is a generic measure of the dispersion of the trajectories whereas *d* and *θ* provide different but complementary information on how the trajectories spread. *d* is linked to the entropy and therefore to the maximum divergence of the trajectories whereas the inverse of *θ* indicates how the trajectories stick together. A high stability implies that only few members strongly deviate from the bulk so that 〈*σ*_*SLP*_〉 is linked to low values of *θ* (in most of the cases). On the rarer cases when most members strongly deviate from each other, we observe a positive correlation between 〈*σ*_*SLP*_〉 and *d* and a weakened link with *θ*. For illustration, we report two examples of both situations in Fig. 5-c (low spread in the ensemble reforecast), and Fig. 5-d (high spread in the ensamble reforecast).

The daily dimension *d* and the inverse of the persistence *θ* therefore have an immediate practical use as proxies for predictability, and could be fruitfully used as a time-effective way in which to evaluate and inform operational forecast systems. For example, one could imagine a system which determines the resolution and ensemble size for a given initialization step based on the values of *d* and *θ*. Moreover, the visualization of the trajectory for a given season (see the supplementary video) can be used to provide a day-by-day tracking of weather extremes.

## Methods

Attractors are geometrical sets that host all the trajectories of a system. To characterize an attractor, one needs to know how often the state *ζ* occurs over a certain time interval and how long the dynamics “sticks” to *ζ* before leaving its neighbourhood. If one is able to specify such properties for all the points of the attractor, then the behaviour of the system is entirely known. The general problem one faces in reconstructing an attractor is the limited number of trajectories that can be observed or simulated^2^. In the case of climate observations we have a single trajectory *x*(*t*) (here represented by the time series of SLP daily fields) that we can exploit to reconstruct the attractor properties.

The purpose of our methodology it is to use a long trajectory *x*(*t*) of system states to reconstruct the salient properties of the attractor. The method is based on the link between extreme value theory (where the extremes are the recurrences of the points *ζ* with respect to all the possible states of the system) and the Poincaré theorem of recurrence. The idea is that each state of the system *x*(*t*) approximates a point *ζ* on the attractor and its neighbours are all the states whose distance with respect to *x*(*t*) is small. So at each time *t* and for each state *x* observed we can define istantaneous properties: the instantaneous dimension *d* and the inverse of the persistence *θ*. The only requirement for the application of the theory is that *x*(*t*) is sampled from an underlying ergodic system. For the theoretical details, demonstrations and examples on dynamical systems see ref. 4. The above properties are instantaneous because they change at each instant *t*, but they are also local, because states observed at different times but close in the phase space will have similar instantaneous properties. We refer to instantaneous dimension rather than to local dimension to avoid ambiguity with the notion of local to indicate a geographic region.

### Instantaneous Dimensions

The distribution of instantaneous dimensions of the attractor of a dynamical system gives useful information on the predictability of observed states because it is related to the Lyapunov exponents^5^. Therefore, estimating the dimension distribution in phase space helps characterizing the overall dynamics of the system. The embedding methods developed in the 1980’s^20^,^21^ do not provide instantaneous dimensions but only the average dimension of the attractor. Moreover, such computations have proved to be problematic in systems with large numbers of degrees of freedom and have given controversial results when applied to atmospheric flows^2^,^22^.

The method we adopt in the present study characterises the points on the attractor by parameters of extreme value laws: if one fixes an arbitrary point *ζ* on a chaotic attractor and considers the probability *P* that a trajectory *x*(*t*) returns within a sphere of radius *ε* centered on the point *ζ*, then the Freitas-Freitas-Todd theorem^23^ modified in ref. 24 states that such probability is a generalized Pareto distribution^25^. The time series of the distance between *ζ* and the other observations along the trajectory is defined by:


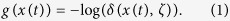


*δ*(*x, y*) is a distance function between two vectors, which tends to zero when *x* and *y* are close to each other. Taking the logarithm increases the discrimination of small values of *δ*(*x, y*) which, as described below, correspond to large values of *g*(*x*(*t*)). The probability of logarithmic returns can then be expressed as:


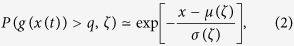


namely an exponential law whose parameters *μ* and *σ* depend on the point *ζ* chosen on the attractor. Remarkably, *σ*(*ζ*) = 1/*d*(*ζ*), where *d*(*ζ*) is the dimension around the point *ζ*. This result has been proved theoretically and verified numerically in several studies collected in ref. 4. In the above equation, *q* is a high threshold, and is linked to the radius *ε* via *q* = *g*^−1^(*ε*) = exp(−*ε*). In other words, requiring that the trajectory falls within a sphere around the point *ζ* is equivalent to asking that the series of *g*(*x*(*t*)) is over the threshold *q*, which can be simply set as a percentile of the series itself. If this approach is iterated for several different *ζ* points, the attractor dimension is then obtained as:


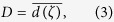


where the overbar means averaging over all *ζ*. This is a powerful result because it provides a direct way to compute dimensions on the attractor without the need for embedding.

### Persistence in phase space

The previous results hold when the state *ζ* being considered is not in the vicinity of a fixed point of the attractor. Fixed points are such that *x*(*t* + 1) = *x*(*t*), for all *t*, i.e. the system is stuck in the same state for an infinite time. In most natural systems, fixed points are unstable: a trajectory passing close to a fixed point spends a finite amount of time in its vicinity before leaving. Such time can be computed by introducing a further parameter in the previous law. This parameter, known as extremal index, is indicated with *θ* and is such that:


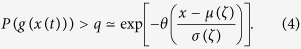


We can interpret *θ* as the inverse of the mean residence time within the sphere. Since 0 < *θ* < 1, low values correspond to high persistence of the trajectory in the neighbourhood of *ζ*, values close to 1 imply that the trajectory immediately leaves *ζ*. The value of *θ* is estimated by using the Suveges likelihood estimator^26^.

### Some idealized examples

We illustrate the procedure to compute the instantaneous dimension described above by applying it to the Lorenz system^7^. This system and its attractor (often referred to as the Lorenz butterfly) have been studied extensively in the literature, and therefore allows us to compare the results of our approach to those of standard techniques in dynamical systems analysis. We begin by generating a trajectory *x*(*t*), using a time step of 0.025. Next, we select approximately 75,000 locations along the trajectory as our *ζ* points on the attractor.

For each *ζ*: (i) the series *g*(*x*(*t*), *ζ*) is computed, ii) a high threshold *q* is selected (here the 98th percentile of the series *g*), (iii) a Generalized Pareto distribution is fitted to the observations exceeding the threshold *q*, iv) an instantaneous dimension of the attractor *d*(*ζ*) is then obtained. Extended Data Fig. A.1 a) displays the values of the instantaneous dimension at all points along our trajectory, while panel (b) displays the corresponding histogram. It is interesting to observe that the minima and maxima of the instantaneous dimension track the extremes of the Lorenz attractor^7^. Maxima of the dimension have a non-trivial structure and are found where recurrences are rare—namely in the wings of the butterfly—and where the trajectories diverge the most—namely between the two wings. The minima correspond to the centre of the butterfly wings, i.e. the fixed points of the Lorenz 63 system. The average value of all the *d*(*ζ*) is, by definition, the attractor dimension *D*. The value we find: *D* = 2.06, corresponds exactly to the value proposed by Grassberger and Procaccia^27^. For this specific example, any *q* larger that the 95th quantile of *g* yields the same results. The persistence, proportional to the inverse of *θ* is also shown in Extended Data Fig. A.1. The Lorenz attractor consists of three unstable fixed points: two at the center of the wings and one at the origin of the axes. The three points are well captured by the statistics of *θ*.

The embedding methodologies adopted in the 1980s were unable to estimate high attractor dimensions^2^, thus providing artificially low values for complex systems. To verify that our methodology does not suffer from the same bias, we have applied it to a range of test fields of the same grid-size as the NCEP data set (Extended Data Section B).

### Sea-level pressure data

In this study we adopt sea-level pressure (SLP) as the meteorological variable to describe the North Atlantic circulation. The major modes of variability affecting the North Atlantic are often defined in terms of the empirical orthogonal functions of SLP^11^,^13^, and a wealth of other atmospheric features, ranging from teleconnection patterns to storm track activity to atmospheric blocking can be diagnosed from the SLP field^16^,^28^.

We base our study on NCEP/NCAR reanalysis data^8^ over the period 1948–2015, with a horizontal resolution of 2.5° and ERA-Interim data^9^ over 1979–2011 with horizontal resolutions of 0.75° and 1.5° respectively. We consider a domain spanning the North Atlantic and Europe (80°W ≤ Long. ≤ 50°E, 22.5°N ≤ Lat. ≤ 70°N). Further tests show that the results are linearly insensitive to the exact boundaries chosen (Extended Data Fig. A.10).

For the NAO, we use daily values computed by NCEP’s Climate Prediction Center. The values are based on NAO patterns which vary on a monthly basis, and cover the full year. The data is freely available from: http://www.cpc.ncep.noaa.gov/products/precip/CWlink/pna/nao.shtml.

### Statistical significance and robustness of the results

For general problems where the value of *D* is not known a priori, the appropriateness of the value of *q* for a given *ζ* can be tested using a number of statistical approaches. In the present study we use the Anderson-Darling test^29^ to test the hypothesis that *g*(*x*(*t*), *ζ*) > *q* come from a generalized Pareto distribution. The test can be repeated for each *ζ* to obtain a statistical significance level for the chosen *q*. All the results displayed in the paper use *q* = 0.98, which satisfies the Anderson-Darling test at the 0.05 significance level for more than 95% of the chosen *ζ*. We further performed a visual inspection of the results, and found them to be stable for 0.99 ≥ *q* ≥ 0.975. In fact, if too high a threshold is selected, the number of values exceeding it is insufficient to successfully fit the generalized Pareto distribution.

**Figure 1:**
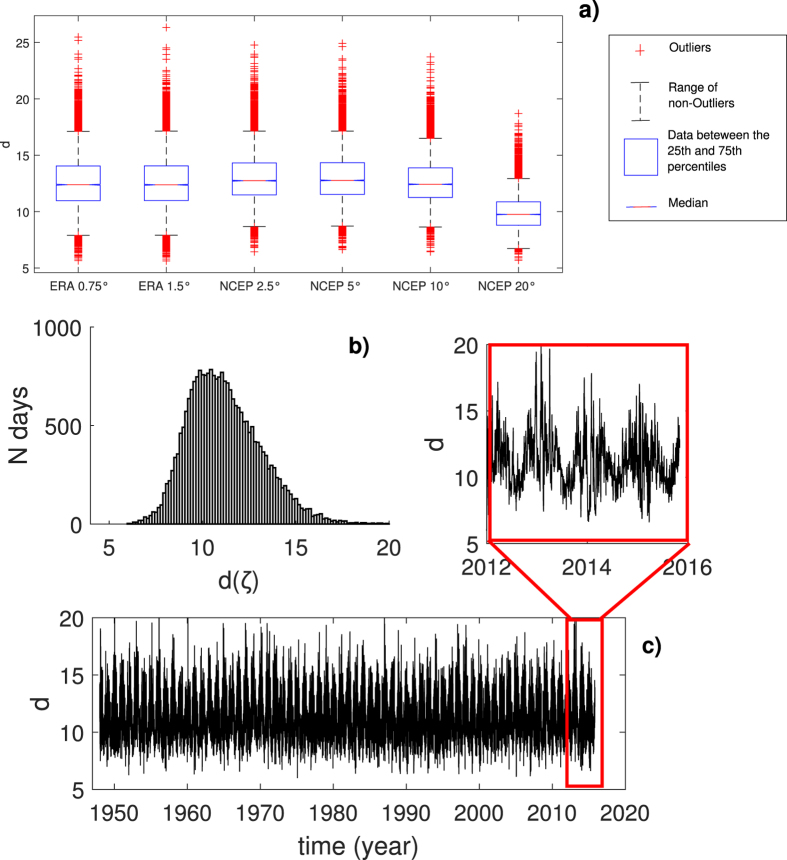
Analysis of the distribution of instantaneous dimensions. (**a**) Box-plots of the distribution of *d*(*ζ*) for different resolutions (in degrees of longitude and latitude). In each box, the central mark is the median, the edges of the box are the 25^th^ and 75^th^ percentiles, the whiskers extend to the most extreme data points not considered outliers and outliers are plotted individually. (**b**) Histogram of the instantaneous dimension *d*(*ζ*) for the NCEP reanalysis. (**c**) Time series of the instantaneous dimensions and inset showing the last 3 years.

**Figure 2:**
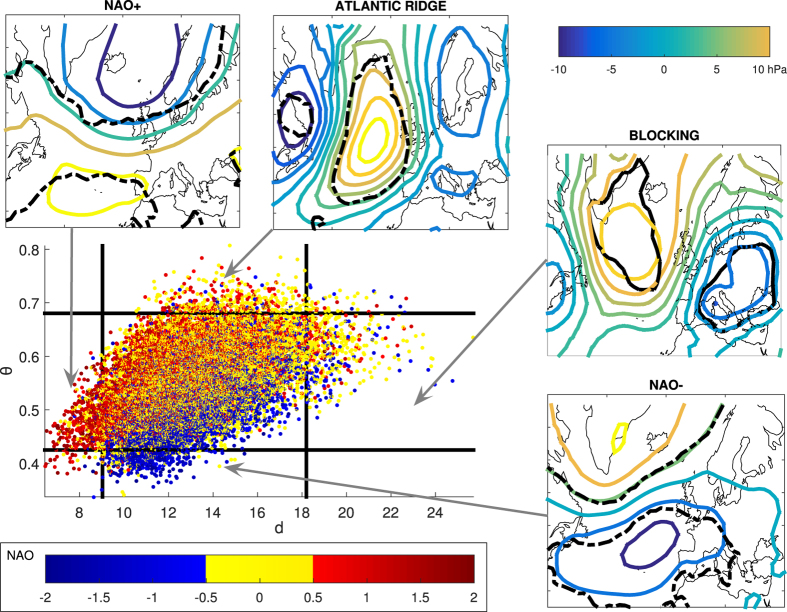
Dynamical systems analysis for the NCEP reanalysis. The scatter plot displays the daily values of the instantaneous dimension *d* and the persistence *θ* of the field. The NAO value for that day is indicated by the colourscale. The black solid lines mark the 0.02 and 0.98 quantiles of *d* and *θ*. The composite anomalies in SLP for the four regions delimited by the black lines are plotted as side panels and can be associated with known weather regimes: NAO+ (minima of *d*), NAO- (minima of *θ*), Atlantic Ridge (maxima of *θ*), Blocking (maxima of *d*). The black lines indicates regions where at least the 2/3 of extreme pressure anomalies have the same sign. The maps in this figure are generated by MATLAB R2013a with M_Map (a mapping package, http://www.eos.ubc.ca/rich/map.html).

**Figure 3:**
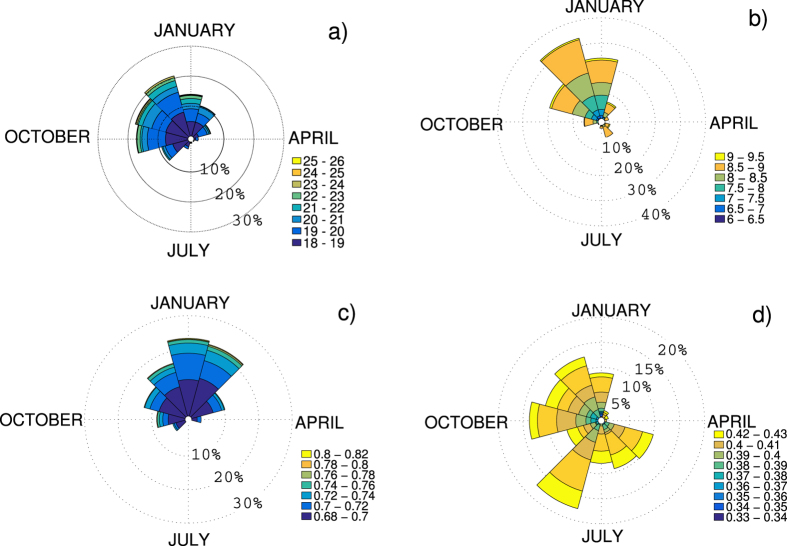
Monthly distribution of the instantaneous properties exceeding the 0.02 and 0.98 quantiles of their respective distributions. Maxima of (**a**) *d* and (**c**) *θ* and minima of (**b**) *d* and *θ* (**d**) for the NCEP reanalysis. The percentage values indicate the occurrences in each month. The colourscale refers to the values of the quantities.

The results presented in Figs 1, 2 and 3 in the main text for the NCEP/NCAR data have been repeated for the ERA-Interim reanalysis, and are shown in Extended Data Figs A.3, A.4 and A.5. Figure A.3 displays the results for both the 0.75° and 1.5° resolutions. It can be seen that both the histograms and the seasonal variability of the instantaneous dimensions are very similar across the three data sets.

### Storm database

**Figure 4:**
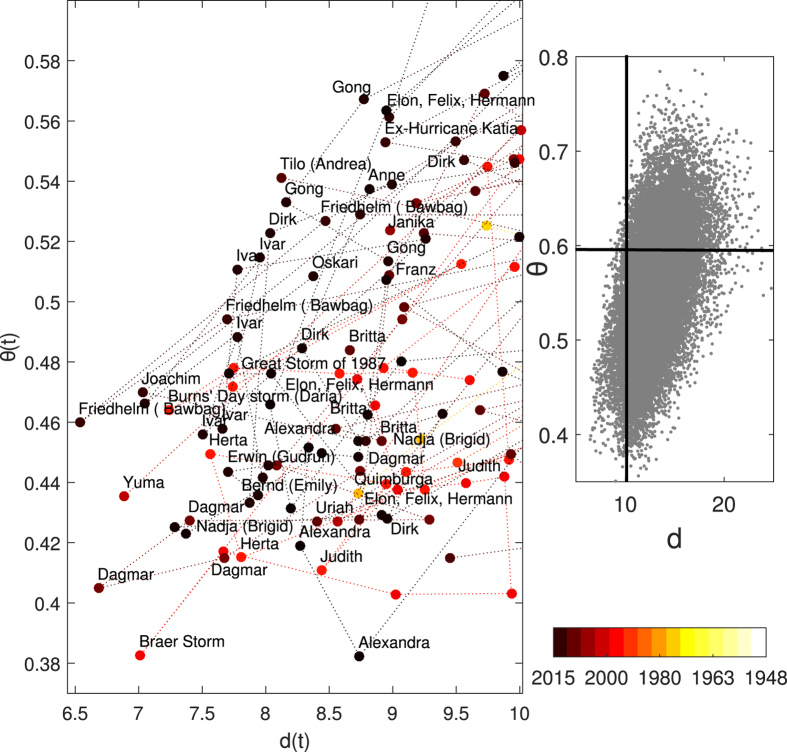
Storms matching the minima of the instantaneous dimensions. The instantaneous dimensions *d* (x-axis) and persistence *θ* (y-axis) for the selected historical storms are plotted along with the storms’ names and years of occurrence (colourscale). Repeated names indicate storms which persisted for several days (see supplementary storm database). The inset shows the full distribution of *d, θ* values. The black lines delimit the phase-space region in which the selected storms lie.

We present a database of historical storms (see Supplementary Information) which affected Europe between 1948 and 2015. This database is largely based on the Lamb^30^ and the Roberts *et al*.^31^ catalogues. Additional storms have been integrated because of their relevance in terms of human losses, damages or their profile in the media. This results in a total of 336 storm dates and 73 distinct storms or storm clusters. The database is organized in four columns: 1) the day of occurrence in the format yyyymmdd, 2) the name(s) of the storm(s), 3) the countries or the region affected and 4) a reference to a peer-reviewed article, a report or a press article describing the importance of the storm. As a caveat to our methodology, we note that the increasing coverage of both meteorological instruments and technological means of information results in an increasing number of recorded storms with time (whereas the minima of the instantaneous dimensions are equally distributed over time). This results in most of the events shown in Fig. 4 occurring in the final part of the NCEP/NCAR reanalysis period.

We use the database to evaluate the correspondence between the minima of the instantaneous dimensions, which equates to positive NAO-like SLP anomalies, and storminess. A day is said to match a storm if it falls within 2 days of the date specified in the database. This accounts for the fact that the recorded dates are typically those when the storm made landfall and/or caused maximum damage, as opposed to the genesis or peak growth phases. 73 of the storm days recorded in our database (or 22%) and 31 individual storms or storm clusters (or 43%) match one of the 481 instantaneous dimension minima (corresponding to the quantile 0.02) identified in the NCEP/NCAR data. This is a very high percentage: if 336 random days are selected (from the extended winter period over which the storms occur), on average only 7 will match an instantaneous dimension minimum. Examples of well-known storms matching low dimensional extremes include Dirk and Herta (see Fig. 4).

### Temperature, precipitation and 10 m wind extremes

Weather regimes can explain a large part of the statistical distribution for surface variables, and have been linked to anomalies in the frequencies of extreme weather events^12^. Here we show that the extremes in phase space we discuss in the main text have a direct link to the occurrence of extreme weather events. At the same time, we note that there are some differences relative to previous analyses based on the traditional weather regimes.

We define extreme weather events as episodes which exceed the 0.98 quantile of the distribution of the anomalies for each gridbox and variable during an extended winter season (September-April). This matches the period of occurrence of the vast majority of the phase space extremes. We then assess the changes in the probability of extreme event occurrences as a function of high and low dimension and persistence in phase space. We build four new distributions, two for high *d*(*ζ*) and *θ* and two for low *d*(*ζ*) and *θ* days. We then compute new percentages of days in each of the four distributions at each gridbox that exceed the original 0.98 quantile thresholds and compare them to the climatological value of 2% probability of occurrence.

Extended Data Fig. A.6 displays the relative changes in the frequency of extreme cold events for (a) high and (d) low *d*(*ζ*). High dimensions show enhanced cold spell frequencies over Scandinavia and northern Europe and the Mediterranean basin. The large signal over Scandinavia is not typically associated with blocking^12^, and is possibly linked to the more eastern location of the high in our pattern. The low dimensions show very large increases in cold extremes over the western North Atlantic and Greenland and south-eastern Europe, matching closely the temperature anomaly footprint of the positive NAO. The link with rainfall extremes (Extended Data Fig. A.6-b,e is less clear. For the high dimensions, significant changes in Europe are limited to regional decreases in extreme wet days over the British Isles and Scandinavia. The low dimensions display instead regional increases over Scandinavia and continental Europe. Finally, the 10 m wind shows a robust dipole pattern, with high dimensions (c) showing more frequent extremes over the Mediterranean region and decreased occurrences over the British Isles and Scandinavia, linked to the blocking high seen in the SLP composites. The low dimensions (f) show an approximately inverse pattern.

Extended Data Fig. A.7 displays the relative changes in the frequency of extreme cold events for (a) high and (d) low *θ*. High *θ* events correspond to frequency increases over the Mediterranean region, in agreement with a similar analysis by Cassou^32^. The low *θ* match instead a strongly enhanced likelihood of cold extremes over the British Isles and Scandinavia, similarly to the impact of the negative NAO phase. Extended Data Fig. A.7-b,e shows the results for wet extremes, which again display less significant links. The most robust feature over Europe is a decrease in the frequency of extreme wet days over the British Isles and Northern France for low *θ* values. In contrast, previous analyses have highlighted the role of a positive NAO in driving wet extremes over these areas^12^,^32^. The 10 m wind displays the largest anomalies over the central and western portions of the domain, with high *θ* (c) showing less frequent extremes over the British Isles, France and the Iberian peninsula and low theta (f) showing a pattern consistent with a more southerly than usual location of the jet stream, associated with a negative NAO^33^.

## Additional Information

**How to cite this article**: Faranda, D. *et al*. Dynamical proxies of North Atlantic predictability and extremes. *Sci. Rep.* **7**, 41278; doi: 10.1038/srep41278 (2017).

**Publisher's note:** Springer Nature remains neutral with regard to jurisdictional claims in published maps and institutional affiliations.

## Supplementary Material

Supplementary Video

Supplementary Information

Supplementary Dataset 1

## Figures and Tables

**Figure 5:**
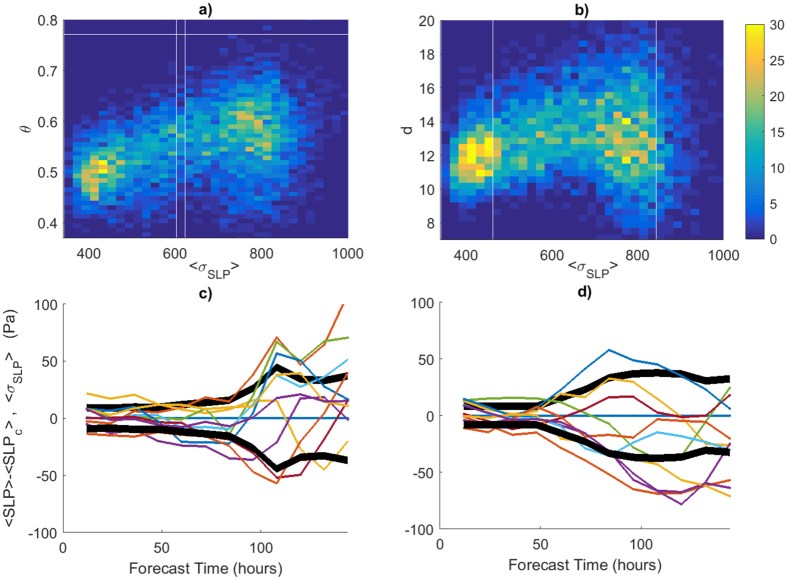
Analysis of the relation between instantaneous properties and NOAA GER reforecast. (**a**,**b**) bivariate histograms of the ensemble spread 〈*σ*_*SLP*_〉 at a lead time of +384 h as a function of the stability *θ* (**a**) and the instantaneous dimension *d* (**b**) of the initialisation field, for the period 2000–2015. The colourscale indicates the number of days with the same pair of parameters. (**c**,**d**) Case studies of how the reforecast trajectories diverge from the control run 〈*SLP*_*c*_〉 and their relation with ensemble spread, *d* and *θ*. (**c**) Reforecast for the 21/01/2000 corresponding to high *d* ≃ 18 with a moderate value of *θ* = 0.56. (**d**) Reforecast for the 12/01/2000 corresponding to high *θ* = 0.73 and moderate value of *d* = 14. The thick black lines show ensemble spread, while the thin coloured lines show the deviation of individual ensemble members from the control member (blue).
