# Supplementary material for: Dynamical proxies of North Atlantic predictability and extremes
Source: Sci Rep. 2017 Jan 25;7:41278. doi: 10.1038/srep41278 (PMC5264183; doi:10.1038/srep41278)
Supplement: Supplementary Information [file srep41278-s2.pdf]

Supplementary Information 'Dynamical proxies of North Atlantic predictability and extremes'

Davide Faranda[1], Gabriele Messori[2] & Pascal Yiou[1]

[1]Laboratoire des Sciences du Climat et de l'Environnement, LSCE/IPSL, CEA-CNRS-UVSQ, Université Paris-Saclay, F-91191 Gif-sur-Yvette, France
[2] Department of Meteorology, Stockholm University and Bolin Centre for Climate Science, Stockholm, Sweden

Legend for supplementary video Upper panel: daily sea-level pressure anomaly (in hPa) for the days of winter 2013-2014. Lower panel: daily values of the instantaneous dimension d and the inverse of persistence θ of the field, for the last 10 days. The maps in the video are generated by MATLAB R2013a with M_Map (a mapping package, `http://www.eos.ubc.ca/~rich/map.html`).

A Additional Figures

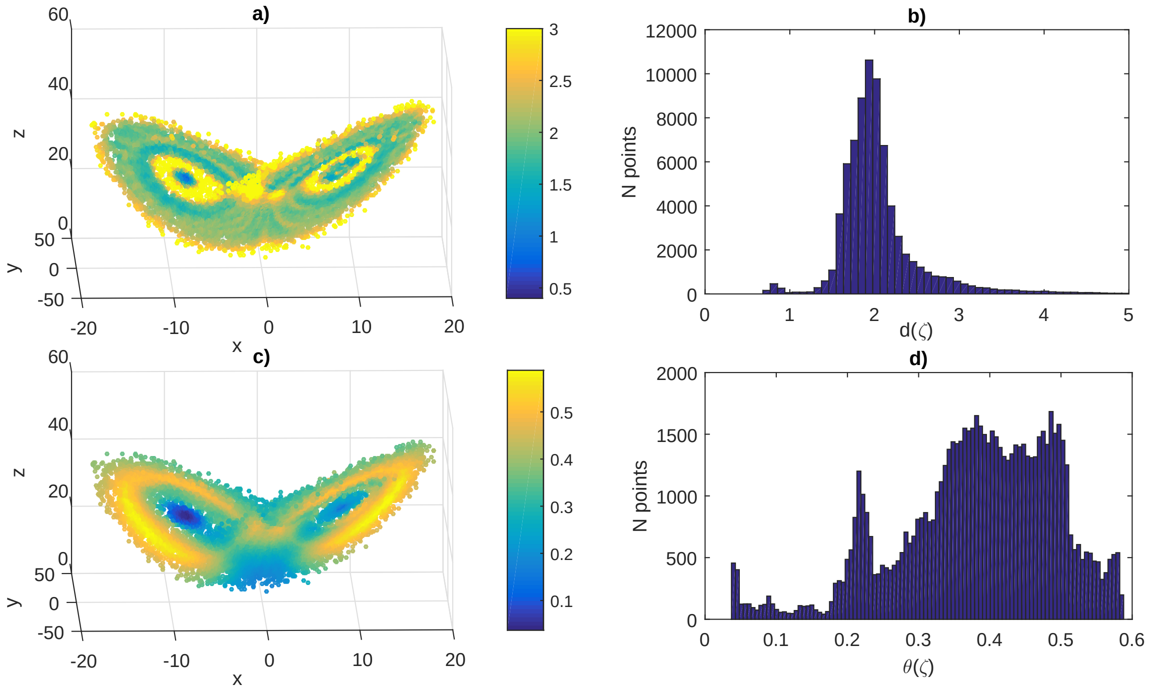

Figure A.1: Test of the algorithm for the Lorenz (1963) attractor. a) Instantaneous dimensions $d(\zeta)$ and b) their histogram, c) inverse of persistence $\theta(\zeta)$ and d) their histogram. All the quantities have been computed at 75,000 ζ points on the Lorenz attractor.

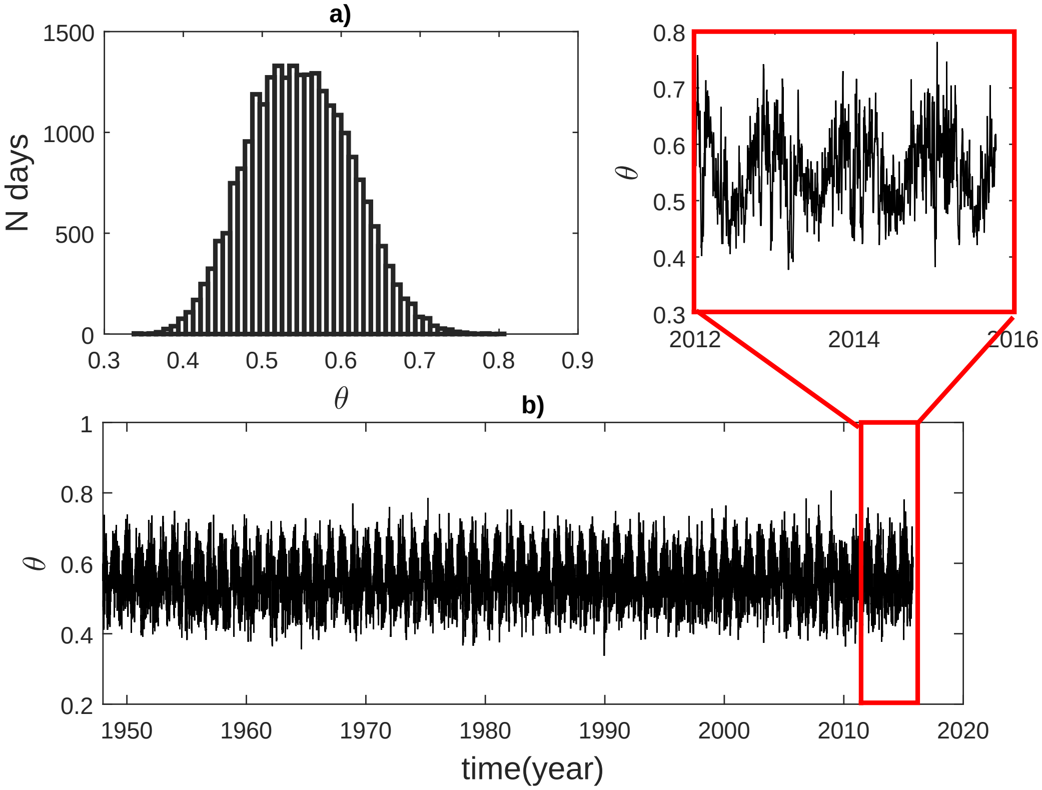

Figure A.2: a) Histogram of the daily inverse of persistence $\theta(\zeta)$ for the NCEP reanalysis. b) Time series of the daily inverse of persistence and inset showing the last 3 years.

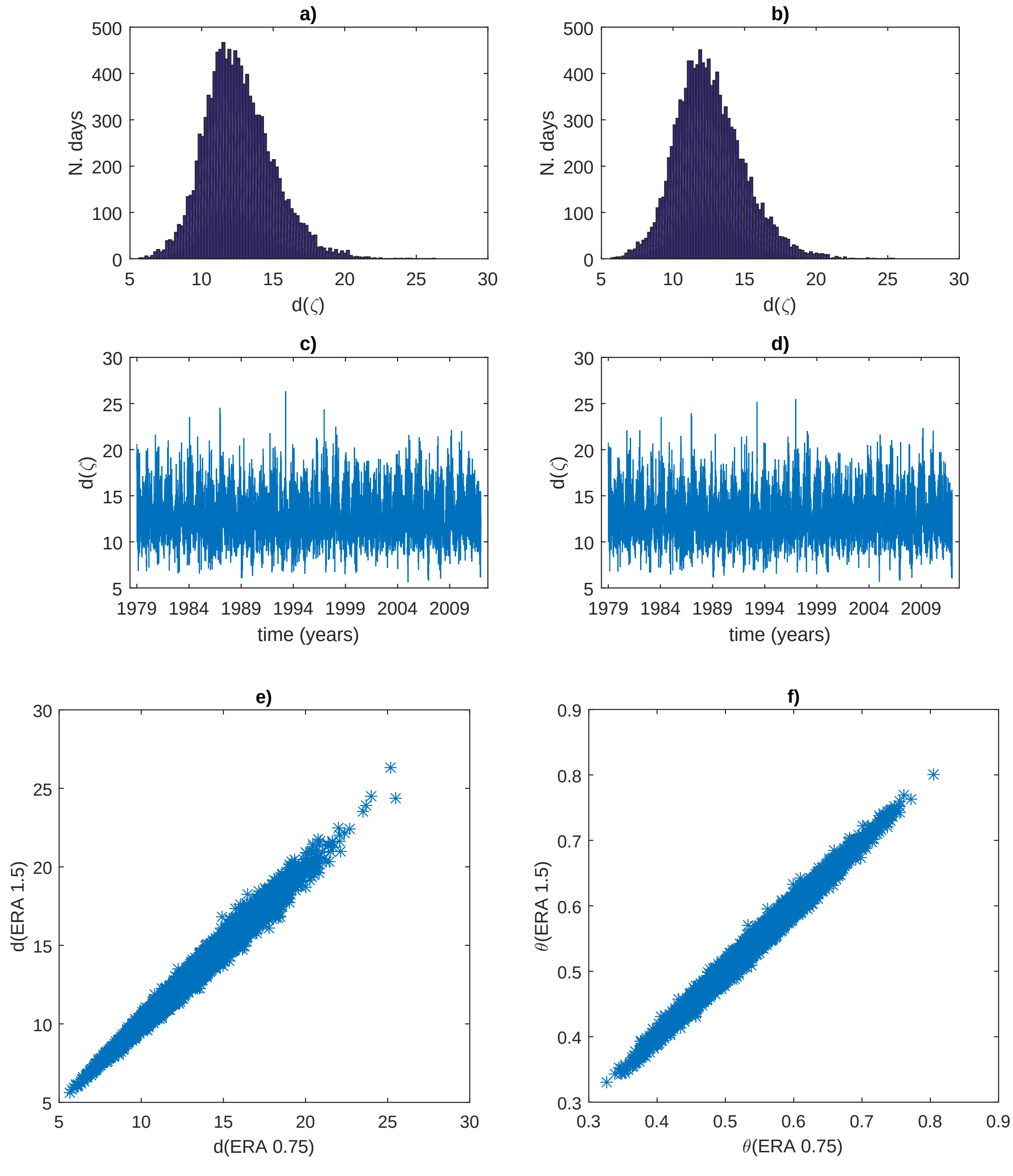

Figure A.3: Histograms of the instantaneous dimension $d(\zeta)$ for a) ERA 0.75 and b) ERA 1.5 data. Time series of the instantaneous dimensions for c) ERA 0.75 and d) ERA 1.5 data. Scatter plots of the values of d e) and θ for the two different resolutions.

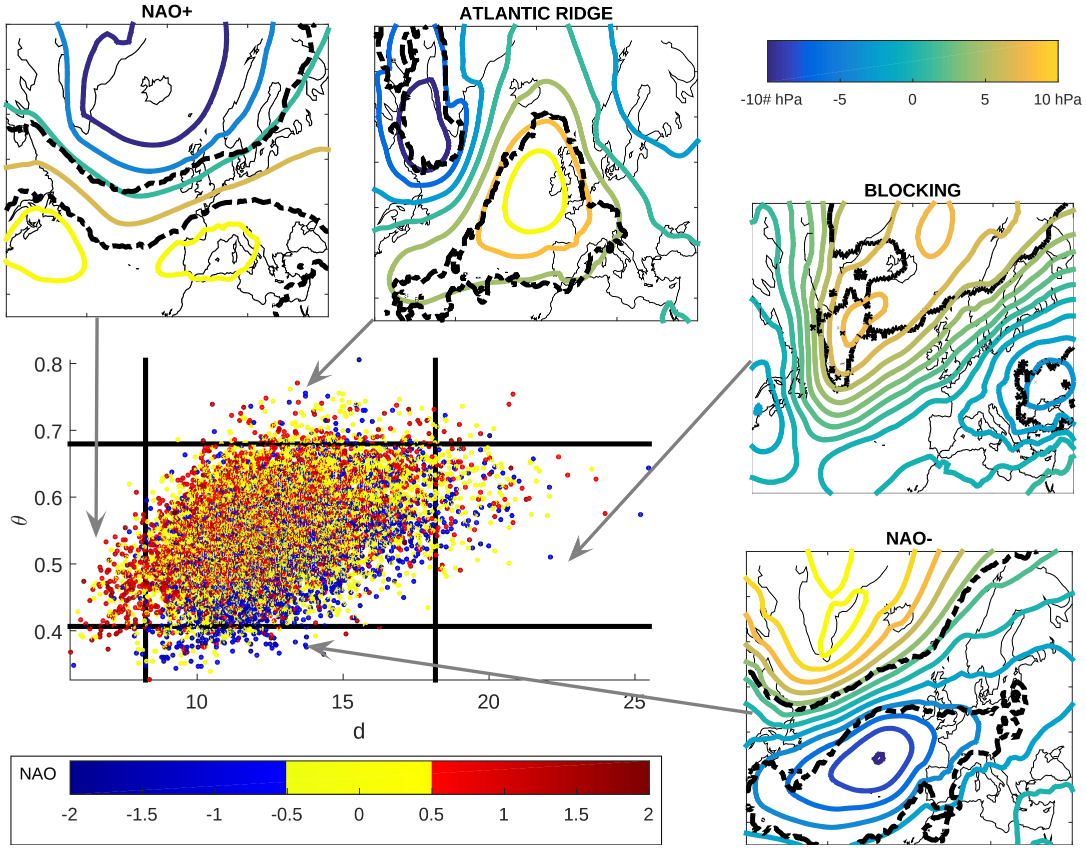

Figure A.4: Dynamical systems analysis for the ERA-Interim data for the period 1979-2015. The scatter plot displays the daily values of the instantaneous dimension d and the inverse of persistence θ of the field. The NCEP-NAO value for that day is indicated by the colourscale. The black solid lines mark the 0.02 and 0.98 quantiles of d and θ. The composite anomalies in SLP for the four regions delimited by the black lines are plotted as side panels and can be associated with known weather regimes: NAO+ (minima of d), NAO- (minima of θ), Atlantic Ridge (maxima of θ), Blocking (maxima of d). The black lines indicates regions where at least the 2/3 of extreme pressure anomalies have the same sign. The maps in this figure are generated by MATLAB R2013a with M_Map (a mapping package, `http://www.eos.ubc.ca/~rich/map.html`).

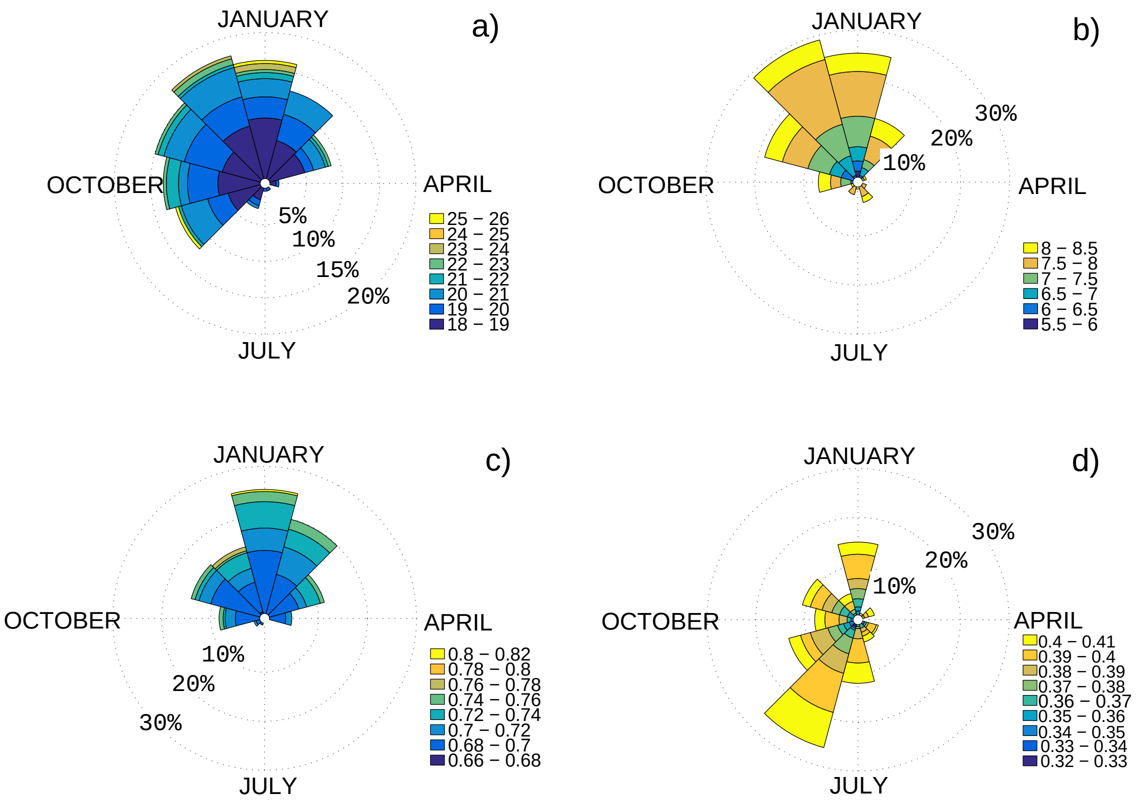

Figure A.5: Monthly distribution of the instantaneous properties exceeding the 0.02 and 0.98 quantiles of their respective distributions. Maxima of a) d and c) θ and minima of b) d and θ d) for the ERA-Interim data for the period 1979-2011. The percentage values indicate the occurrences in each month. The colourscale refers to the values of the quantities.

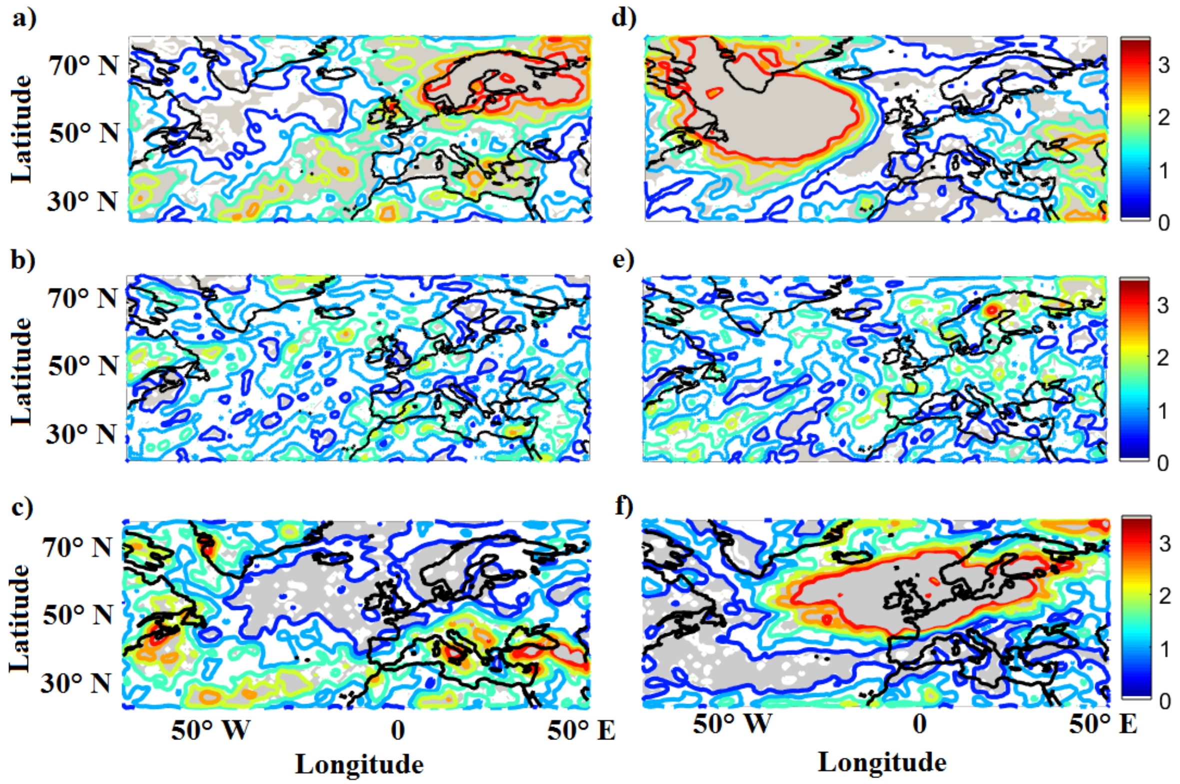

Figure A.6: Relative changes in the frequency of a), d) extreme cold; b), e) extreme wet and c), f) extreme 10m wind events for days with instantaneous dimension beyond the a-c) 0.98 and d-f) 0.02 quantiles of d in the ERA-Interim data. Contours start at 0, with an interval of 0.5; the grey shading shows statistically significant changes. The maps in this figure are generated by MATLAB R2013a.

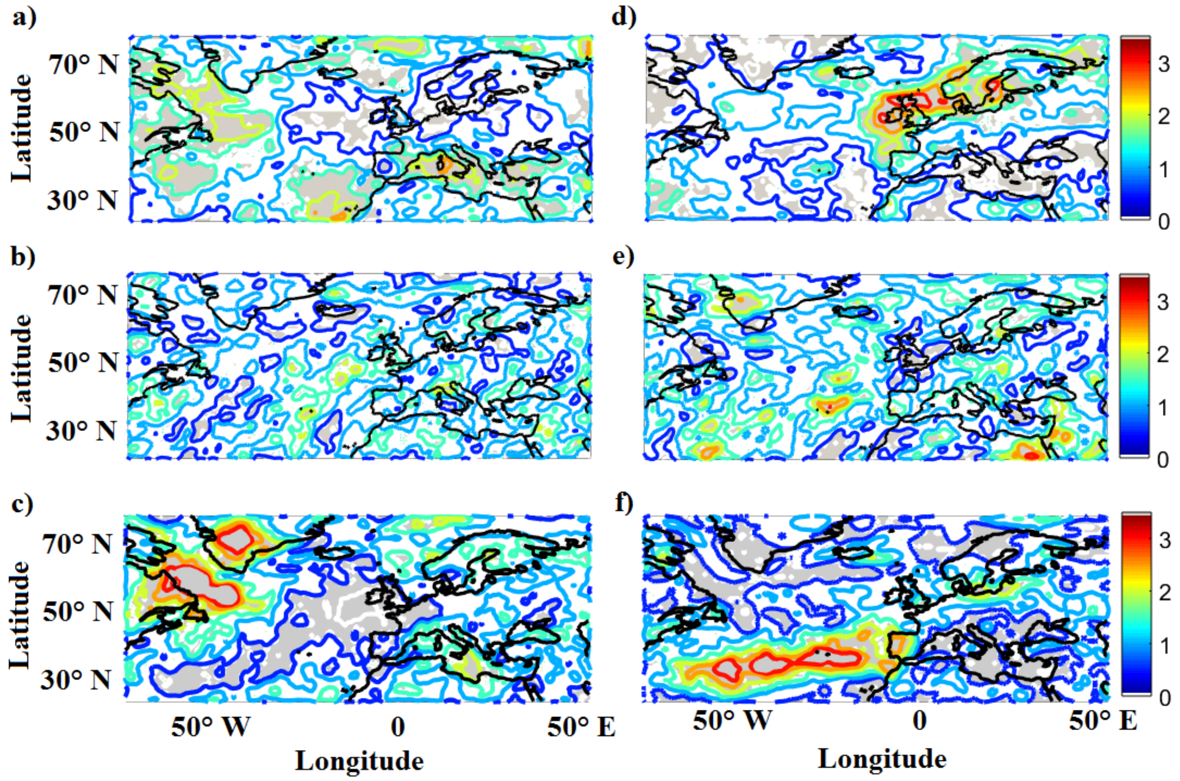

Figure A.7: Relative changes in the frequency of a), d) extreme cold; b), e) extreme wet and c), f) extreme 10m wind for days with inverse of persitence θ beyond the a-c) 0.98 and d-f) 0.02 quantiles of θ in the ERA-Interim data. Contours start at 0, with an interval of 0.5; the grey shading shows statistically significant changes. The maps in this figure are generated by MATLAB R2013a.

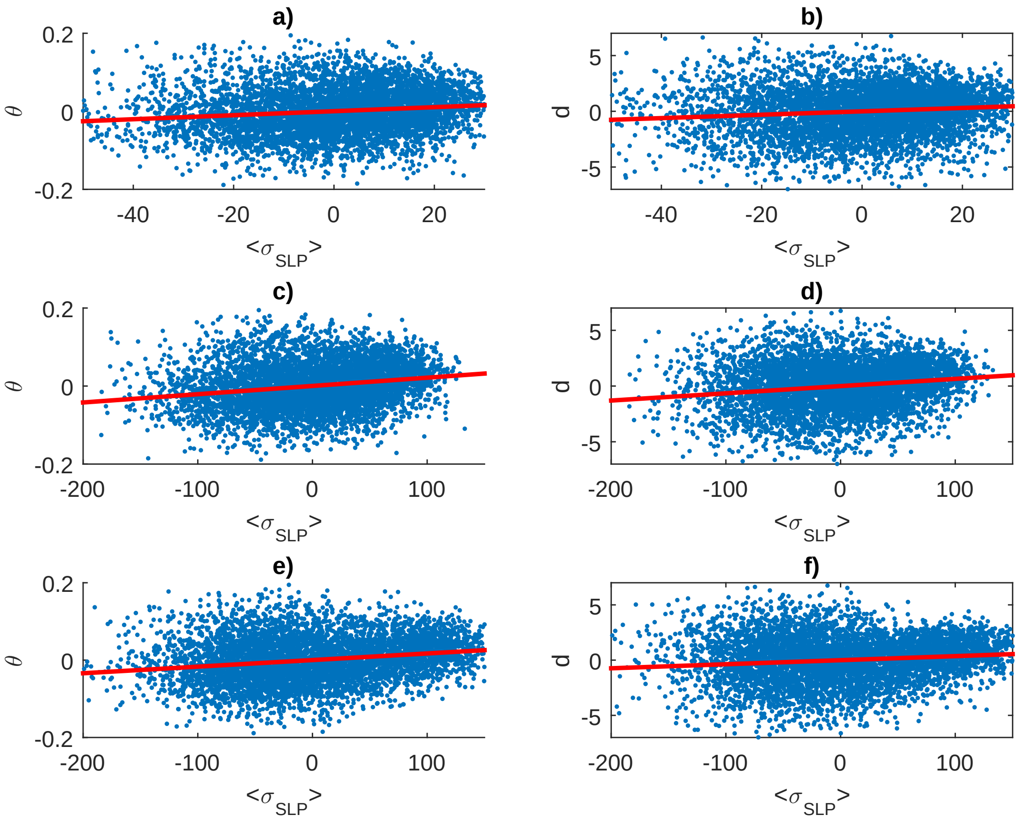

Figure A.8: **Analysis of the relation between instantaneous properties and NOAA GER reforecast** for the anomalies computed with respect to the seasonal cycle, defined using a moving average filter with a window of 180 days. Scatter plots and linear fit of the ensemble spread $\langle\sigma_{SLP}\rangle$ at a lead time of 72h a-b), 192h c-d) and 384h e-f) as a function of a),c),e) the inverse of the persistence θ and b),d),f) the instantaneous dimension d of the initialisation field.

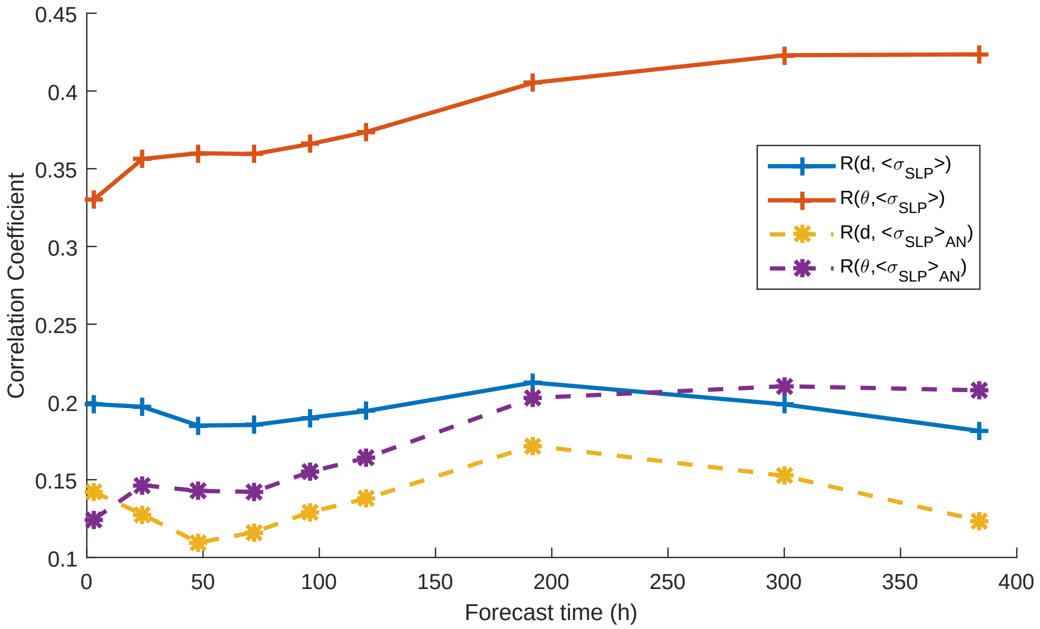

Figure A.9: Correlation coefficient R between the ensemble spread $\langle \sigma_{SLP} \rangle$ and the inverse of the persistence θ (red) and the dimension d (blue) at different forecast times, for the full time series (red, blue) and for the anomalies (yellow, violet) computed with respect to the seasonal cycle defined using a moving average filter with a window of 180 days.

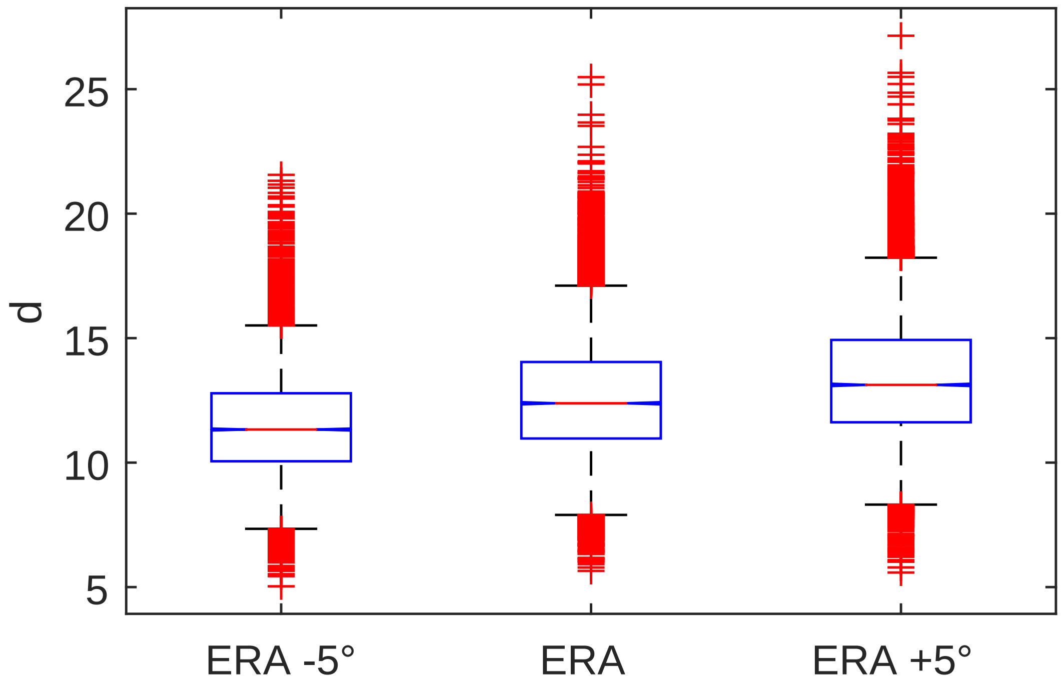

Figure A.10: Box-plots of d for different domain sizes. The region has been shrunk or extended by 5° in longitude and latitude. In each box, the central mark is the median, the edges of the box are the 25th and 75th percentiles, the whiskers extend to the most extreme data points not considered outliers, and outliers are plotted individually.

B Analysis for Synthetic field

The embedding methodologies adopted in the 1980s were unable to estimate high attractor dimensions, thus providing artificially low values for complex systems. To verify that our methodology does not suffer from the same bias, we have applied it to a range of test fields of the same grid-size as the NCEP data set:
i) A series of 50000 completely random fields obtained by generating a $N = 20 \times 20$ matrix of normally distributed random numbers $\mathcal{N}$, renewed at each time steps (function *randn* in MATLAB):

$$z_{i,j}(t) = \mathcal{N}(t; N) \tag{1}$$

ii) a series of 50000 random shifts of a coherent field:

$$z_{i,j}(t) = \mathrm{mod}(z_{i+\xi(t),j+\eta(t)}(t-1), N) \tag{2}$$

where $\{\eta, \theta\}$ are signed integers $\in [-2, -1, 0, 1, 2]$ and are the same for all the $z_{i,j}$ elements of Z, a matrix of size $N = 20 \times 20$ obtained via the function *peaks* in MATLAB:

$$z_{i,j} = 3(1-x)^2 \exp(-(x^2) - (y+1)^2) - 10(x/5 - x^3 - y^5)\exp(-x^2 - y^2) - 1/3\exp(-(x+1)^2 - y^2) \tag{3}$$

the mod operation indicates that the domain is periodic. Such synthetic fields exhibit a complex dynamics that have a dimension that increase with the number of grid points. The exact distribution of instantaneous dimension d for those systems is not known theoretically. However, since the dimension is a measure of the degrees of freedom active in the domain at each instant, one can conjecture that: i) the distribution of d for the random field must be a large fraction of the grid size, ii) the distribution of d for the shifts of the coherent field must be centered around 5, the number of coherent structures used as initial condition in equation 3.
The results reported in Extended Data Fig. B.1 confirm this hypothesis and show that for those random fields (whose dimension is high by construction), we have obtained estimates for D larger than 80. Conversely, estimates of D for the fields with coherent structures resembling pressure centres were lower than 5. This suggests that the methodology we adopt in the present study is able to capture a wide range of instantaneous local dimensions.

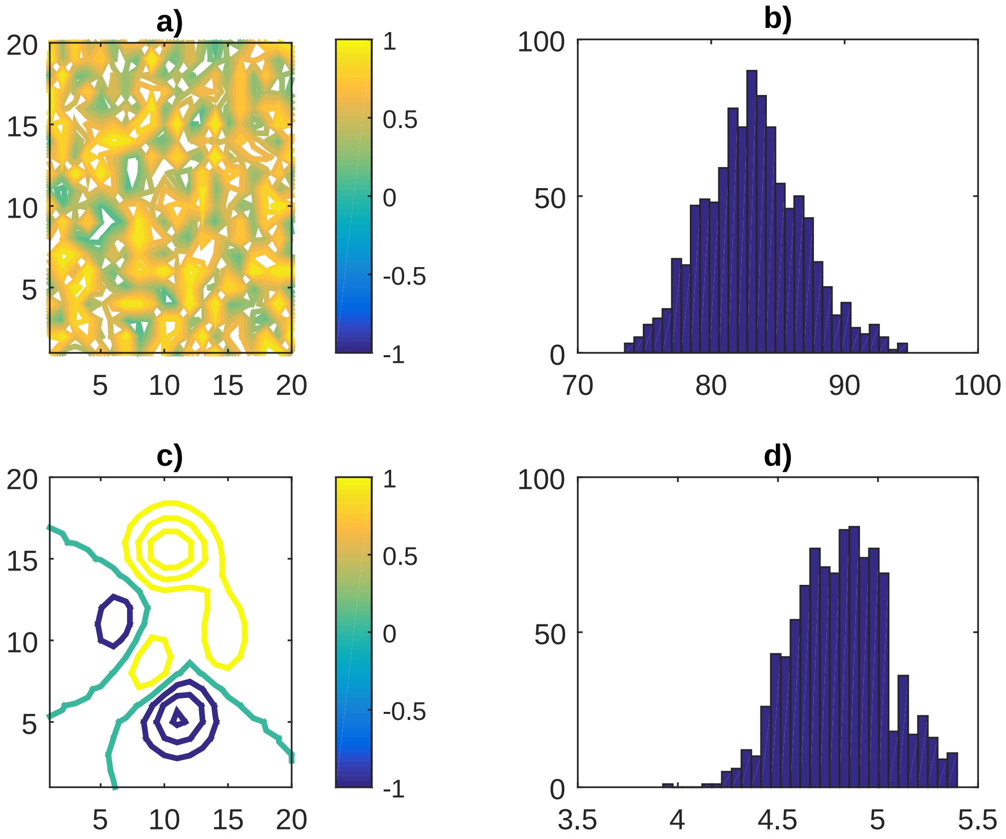

Figure B.1: Test of the algorithm on synthetic fields of size 20×20. The fields have been randomly iterated 50000 times to mimic chaotic dynamics. a) Random field example. b) histogram of the instantaneous dimensions: $D = 83.12$. c) Field with coherent structures resembling pressure fields. d) Histogram of the instantaneous dimension: $D = 4.8$.
